# A Case of Anti-Jo-1 Myositis with Unique Biopsy Findings

**DOI:** 10.1155/2022/9096643

**Published:** 2022-06-06

**Authors:** Beenish Zulfiqar, Pavel Aksionav, Mohamad Bittar, Cathy Chapman

**Affiliations:** Department of Rheumatology, University of Tennessee, Memphis, Tennessee, USA

## Abstract

Antisynthetase syndrome (ASS) or anti-Jo-1 antibody syndrome has a classic clinical presentation including arthritis, myositis, interstitial lung disease, mechanic hands, and/or Raynaud's phenomenon. The biopsy findings are distinctive from polymyositis or dermatomyositis. We describe an interesting case of ASS where a patient presented with significant muscle weakness, proteinuria, and interstitial lung disease. She also had positive Ro-52 antibodies in addition to anti-Jo-1 antibodies. Her biopsy findings were consistent with inflammatory necrotizing myositis.

## 1. Introduction

The antisynthetase syndrome is a rare autoimmune disease characterized by the presence of one of the antisynthetase antibodies and at least one of the following clinical features: inflammatory myopathy, inflammatory polyarthritis, or interstitial lung disease (ILD). The symptoms and severity of the disorder can vary among affected individuals. Other symptoms may include fever, “mechanic's hands,” and Raynaud's phenomenon. Studies have shown that most patients with antisynthetase autoantibodies have ILD. We report the case of a middle-aged female with biopsy-proven inflammatory myopathy and a positive anti-Jo-1 antibody who was found to have new onset proteinuria. Additionally, she had very interesting biopsy findings which showed features of overlap between dermatomyositis and polymyositis. It is critical to maintain a broad differential, which includes unusual manifestations of rare rheumatologic disease. Our patient also had positive Ro-52 antibodies, which is a myositis-associated antibody with the antisynthetase syndrome.

## 2. Case Presentation

**Figure 1:**
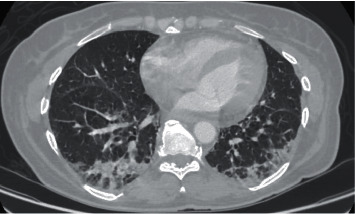
CT chest w/o contrast showing ground-glass opacities, bronchiectasis, and increased interstitial markings.

A 53-year-old Caucasian female with a past medical history significant for iatrogenic hypothyroidism, chronic (>1 year) hoarseness (followed with ENT and treated with PPI, thought to be due to gastroesophageal reflux disease), and a recent episode of pneumonia (she was hospitalized one month prior and treated for community-acquired vs viral pneumonia) presented with generalized muscle weakness, lower leg edema, and bilateral hand tenderness. Review of systems was positive for dysphagia for 1 month which started with liquids and progressed to solid food as well. Physical exam revealed three out of five muscle strength in proximal upper and lower limbs, diffuse muscle tenderness, bilateral metacarpophalangeal joint swelling and tenderness to palpation, and 4+ lower leg edema up to the knees. No pharyngeal redness or discharge was observed. Lab work showed white blood cell count −18.36 10 *∗* 3/*μ*L (ref range 4.0–10.0), ferritin −2122 ng/mL (ref range 5.0–204), lactate dehydrogenase −960 U/L (ref range 125–220), creatine phosphokinase −3261 U/L (ref range 29–168), alanine aminotransferase −198 U/L (ref range 0–55), aspartate aminotransferase −175 U/L (ref range 5–34), sedimentation rate −30 mm/hr (ref range 0–20), C-reactive protein −27.1 mg/L (ref range 0–5), hemoglobin −10.8 g/dL, albumin level −2.1 g/dL, creatinine −0.59 mg/dl (ref range 0.6–1.6), procalcitonin −0.05 ng/mL (ref range 0–0.25), thyroid-stimulating hormone −9.2 *μ*IU/mL (ref range 0.35–4.94), free T4 −0.82 ng/dL (ref range 0.7–1.48), urine protein 2+, no hematuria, no RBC casts, protein/creatinine ratio −774 mg/g, and negative blood and urine culture. CT chest showed ground-glass opacities, bronchiectasis, and increased interstitial markings (Figure 1).

A swallowing study showed gastroesophageal reflux to the inlet with poor clearance, laryngeal penetration without evidence of tracheal aspiration, and mild vallecular pooling. Initial differential diagnosis was broad and included an autoimmune process, a paraneoplastic syndrome, myositis triggered by viral or bacterial infection, and atypical presentation of COVID-19 including multisystem inflammatory syndrome in adults (MIS-A). The case was complex and involved collaboration between different specialties.

The patient is up to date on age-appropriate cancer screening. COVID-19 PCR and antibodies came back negative. The patient was covered with broad-spectrum antibiotics since admission without improvement. Available imaging studies were negative for malignancy. Nephrology was consulted and suspected the rheumatologic cause of new proteinuria. They did not consider a renal biopsy as proteinuria was below nephrotic range with stable kidney function. Pulmonology consulted and suspected rheumatological disease-related interstitial lung disease vs resolving pneumonia from the previous admission. In the setting of progressing muscle weakness and high suspicion for an autoimmune process it was decided to start on steroids empirically.

The patient was started on IV Solumedrol (500 mg IV for two days, decreased to 250 mg IV on the 3rd day) and resulted in clinical improvement. The patient had a marked decrease in wrist swelling, increase in muscle strength, decrease in lower leg edema, and improvement in dysphagia. After 3 days of IV Solumedrol, the patient was started on oral prednisone taper and was discharged home. Lab work showed positive ANA (no titer available), positive anti-Jo-1 166 Units (ref range < 20), positive Ro-52 antibodies 78 Units (ref range < 20), and elevated aldolase 67.8 U/L (ref range 3.3–10.3). Muscle biopsy showed abundant necrotic muscle fibers, many of them undergoing phagocytosis; however, there was no evidence of interstitial or perivascular inflammation. No vasculitis or rimmed vacuoles were seen. Fiber type distribution appears grossly normal. There was evidence of upregulation of the major histocompatibility complex (MHC) in a patchy way, and there were some scattered lymphocytes in the fascia and interstitial area.

Other serological tests, including tests for rheumatoid factors, anti-cyclic citrullinated peptide antibodies, antineutrophil cytoplasmic antibodies, and the rest of the antiextractable nuclear antigen panel, were negative. All other labs including Hb, albumin level, ALT/AST, ferritin, LDH, CPK, and urine protein level normalized or significantly improved.

On a follow-up visit after discharge, the patient had mild muscle weakness in all limbs but was able to walk without assistance; lower leg edema significantly improved; no synovitis was noted on exam. The patient was started on a steroid-sparing agent, Azathioprine 50 mg BID. She was referred to Nephrology to follow up on proteinuria which had significantly improved as well as Pulmonology for interstitial lung disease workup including pulmonary function test and high-resolution CT chest which are pending to date.

## 3. Discussion

Antisynthetase syndrome could fall into either polymyositis (PM) or dermatomyositis (DM), with the triad that includes interstitial lung disease, arthritis, and mechanic's hands. The most common manifestation, however, is the presence of interstitial lung disease, which has been reported to be present in up to 70% of the cases [1]. The most common antibody associated with antisynthetase syndrome is anti-Jo-1 antibody, which is found in about 25–30% of the cases followed by PL-5 (2–5%) and PL-12 (2–5%) [1]. Our patient had a strongly positive anti-Jo-1 antibody and had a positive ANA as well as Ro-52 antibodies. Similar to our case, it has been noted that in addition to myositis specific antibodies, these patients may also have positive myositis-associated antibodies such as ANA, SSA, and PM/Scl antibodies [2]. Anti-Ro-52 is found to be positive in about 37% of patients with PM or DM, and it is associated with severe muscle, joint, and lung involvement. Additionally, it has been observed that these patients are less likely to respond to immunosuppression [2]. In a study published by La Corte et al. [3], the authors compared clinical symptoms and antibody profile in 28 patients with ASS and 48 patients with typical PM and DM. Patients with classic PM and DM had significant skin and muscle involvement while patients with ASS had ILD, mechanic's hands, and arthritis as peculiar features. It was also noted that ASS patients with positive SSA (Ro) antibodies had severe ILD, which was expressed as high-resolution CT score > 7. However, after 3 years of follow-up, the antibody profile in ASS did not affect the prognosis or survival. Our patient, although asymptomatic from the respiratory standpoint, she had chronic opacities in bilateral lungs on imaging which warranted ILD work up.

Our patient's muscle biopsy was consistent with necrotizing inflammatory myositis, with multiple fibers undergoing necrosis and phagocytosis, without much evidence of perifascicular inflammation, with no evidence of myonuclear filament inclusions, which is not what is typically seen in ASS biopsy. In an article published by Mozaffar [4], the authors compared the pathology obtained from muscle biopsies of 11 patients with ASS to other forms of inflammatory myositis. They concluded that patients with ASS with positive anti-Jo-1 antibodies had perimysial inflammation with fragmentation of macrophages which seem to be the dominant cells contributing to the inflammatory process. Patients with severe muscle weakness were found to have atrophy and necrosis as well as regenerating muscle fibers. There was no inflammation noted in endomyseal and perifascicular regions as we may see in other inflammatory myopathies. In another study conducted by Mescam-Mancini et al. [5], they compared muscle biopsy results of 53 patients with ASS to 63 patients with other inflammatory myositis including DM, inclusion body myositis (IBM), and immune mediated necrotizing myositis (IMNM). It was noted that significant perifascicular atrophy is seen in DM while anti-Jo-1 associated myositis has abundant necrosis and complement deposition in peri fascicular region with MHC I expression. It also notably has more inflammatory fibers than atrophied fibers in the perimysium whereas our patient had less inflammation and more atrophied fibers with prominent necrosis. Werner et al. [6] obtained muscle biopsies from 21 patients with ASS. They concluded that ASS-associated myositis is characterized by distinctive myonuclear actin filament inclusions, including rod formation and a typical necrotizing perimysial myositis which supported that ASS-associated myositis is unique and should not be grouped among dermatomyositis, polymyositis, sporadic inclusion body myositis, necrotizing autoimmune myositis, or nonspecific myositis. The study provided Class II evidence that the presence of myonuclear actin filament inclusions accurately identifies patient with ASS-associated myositis with sensitivity of 81% and specificity of 100%. There were no myonuclear actin filament inclusions in our patient's muscle biopsy. Noguchi et al. [7] evaluated histologic findings in 50 patients with ASS. Myofiber necrosis in the perifascicular region was observed in 24 patients (48%). 8 patients (16%) showed diffusely distributed necrotic and regenerating fibers. In other 18 patients (36%), few necrotic and regenerating fibers with perimysial (usually perivascular) mononuclear cellular infiltration and/or MHC class I and II expression on the cytoplasm and sarcolemma of nonnecrotic and nonregenerating fibers were observed. Our patient's biopsy did not show evidence of interstitial of perivascular inflammation.

Interestingly, our patient was found to have an elevated 24-hour urine protein/creatinine ratio, ranging between 0.5 gm and 0.7 gm/24 hours during the hospital stay. She had no active urinary sediments, neither did she have history of acute kidney injury or chronic kidney disease. She did not have proteinuria during her previous hospital visits and had a normal body mass index. In a study conducted by Couvrat-Desvergnes et al. [8], they compared about 150 patients with DM, PM, and ASS, who had renal involvement in the form of acute kidney injury, chronic kidney disease, and proteinuria. They noted that patients with acute kidney injury (*n* = 16, 11%) were predominantly males (*p* < 0.05), had an older age of symptom onset (*p* < 0.05), had diabetes and hypertension at the time of disease onset (*p* values 0.02 and 0.04, respectively), had less muscle involvement (calculated as mean muscle scores (*p*0.04), and initial proteinuria defined as 0.3 g/day (*p* < 0.05). Similar features were noted in patients with chronic kidney disease. A few of the study subjects underwent a renal biopsy. Biopsy results varied greatly and included the following pathologies: “edematous thickening of the intima of an interlobular artery superimposed on chronic arteriosclerosis; marked fibrous thickening of the intima of an arcuate artery; cresentic glomerulonephritis with negative ANCAs; IgA nephropathy” to mention a few. Our patient did not get a renal biopsy during her hospital stay. On follow-up visit in 8 weeks with Rheumatology, her proteinuria had resolved on urinalysis. While we do not have strong evidence to support that the patient's proteinuria was caused by the underlying inflammatory process, it was a new finding and resolved with the treatment of myositis, which may point towards its association with inflammatory myositis.

## 4. Conclusion

1. ASS may not follow the classic triad of ILD, mechanic's hands, and arthritis. In some cases, like ours, muscle weakness may take precedence.
2. ILD may be asymptomatic as in our case and needs extensive work up with careful monitoring, especially in the presence of SSA antibodies.
3. Biopsy findings in ASS may not be typical and may not fall into either PM or DM categories.
4. Some patients might have renal involvement. A thorough work up including renal biopsy might be helpful in certain cases.

## Data Availability

The data supporting literature relevant to the case have been cited in the text under ‘References.'

## Additional Points

*Limitations*. Our patient did not get a renal biopsy; hence, we could not establish an etiology for her proteinuria. We do not have a picture of a muscle biopsy (only lab report). Additionally, we do not have studies such as pulmonary function tests, urine protein creatinine ratio prior to her hospital admission to establish comparison with recent findings. We also do not have the pulmonary function tests after hospital discharge due to loss of follow-up.
